# Proteoliposome Engineering with Cell‐Free Membrane Protein Synthesis: Control of Membrane Protein Sorting into Liposomes by Chaperoning Systems

**DOI:** 10.1002/advs.201800524

**Published:** 2018-08-23

**Authors:** Mitsuru Ando, Shun Schikula, Yoshihiro Sasaki, Kazunari Akiyoshi

**Affiliations:** ^1^ Department of Polymer Chemistry Graduate School of Engineering Kyoto University Katsura, Nishikyo‐ku Kyoto 615‐8510 Japan; ^2^ Japan Science and Technology Agency (JST) The Exploratory Research for Advanced Technology (ERATO) Bio‐Nanotransporter Project Katsura Int'tech Center Katsura, Nishikyo‐ku Kyoto 615‐8530 Japan

**Keywords:** cell‐free protein synthesis, hexahistidine‐fused membrane proteins, nickel‐chelating liposomes, polyhistidine/nickel‐chelate affinity, proteoliposomes

## Abstract

Integral membrane proteins (IMPs) modulate key cellular processes; their dysfunctions are closely related to disease. However, production of IMPs in active conformations for further study is hindered by aggregation and toxicity in living expression systems. IMPs are therefore produced in cell‐free systems employing liposome chaperoning, but membrane integration of the nascent IMPs is suboptimal and orientation of the integrated proteins remains uncontrollable. Thus, an artificial membrane protein sorting system is developed, based on polyhistidine/nickel‐chelate affinity, combined with cell‐free membrane protein synthesis. Its proof of concept is demonstrated with a N‐terminal hexahistadine‐fused conexin‐43 (NHis–Cx43) model IMP. Nickel‐chelating liposomes efficiently incorporate twofold newly synthesized NHis–Cx43 compared with Cx43. NHis–Cx43, when synthesized in this system, forms dye‐permeable hemichannels, similar to plasma membrane pores formed by Cx43 in cells. The topology of incorporated NHis–Cx43 indicates two orientations in the liposomal membranes. However, NHis–Cx43 orientation is controlled, resulting in single topology, by combination of the natural molecular chaperone DnaKJE. Successful synthesis and at least 4.5‐fold increase lipid incorporation are also achieved with three other NHis‐fused IMPs, including α‐helix and β‐barrel IMPs. Overall, this simple membrane protein sorting system is usable combined with molecular chaperones to prepare proteoliposomes for many applications.

## 1 Introduction

Integral membrane proteins (IMPs) modulate processes involved in cellular homeostasis, such as signal transduction, ion transport, and cellular communications, through their substrate specificities.^1^ In addition, IMP dysfunctions were closely related to onset of certain diseases.^2^ Therefore, elucidating structure and function of IMPs should accelerate understanding of biological phenomena, construction of advanced nanodevices,^3^ drug discovery,^4^ and artificial cells.^5^ However, isolation of IMPs with active conformations remains challenging because hydrophobic IMPs form irreversible aggregates in water. Furthermore, overexpressions of IMPs are toxic in transgenic living systems, resulting in low yields of target IMPs.^6^

Cell‐free protein synthesis systems are customizable transcription/translation systems in test tubes, using cellular extracts^7^ or reconstituted transcription or translation related factors (PURESYSTEM).^8^ This unique concept enables preparation of proteins under conditions not compatible with living systems, including in the presence of detergents, lipids, or cellular toxic compounds. Liposome chaperoning technology was developed to obtain IMP‐reconstituted liposomes (proteoliposome), that is, with IMPs synthesized by cell‐free protein synthesis in the presence of liposomes.^9^ Notably, after mixing liposomes with cell‐free protein synthesis reagents, newly formed IMPs are spontaneously integrated into liposomal membranes. In previous studies, we demonstrated that integrated IMPs were spontaneously oligomerized into their active forms and exhibited their expected functions.[[qv: 9b‐e]] Furthermore, over 90% of *Escherichia coli*‐derived aggregation‐prone 85 IMPs were solubilized by this liposome chaperoning technology.[[qv: 9f]] However, these previous studies also showed that this technology did not provide sufficient integration efficiency for all IMPs and the topology of the integrated IMPs remained unclear.

In living systems, the fate of nascent proteins is determined by several molecular machineries, such as chaperone, translocon, and proteasome systems. In particular, the signal recognition particle (SRP)/SRP receptor (SR)–translocon pathway is a well‐known sorting modulator for nascent proteins. The hydrophobic signal peptide or transmembrane domain of nascent proteins, generating from ribosomes, is recognized and protected by SRP and directed toward the endoplasmic reticulum membrane, resulting in safe sorting of the signal peptide or transmembrane domain of proteins into membranes.^10^ Several studies demonstrated that SecY/SecE/SecG translocon‐reconstituted proteoliposomes supplemented with other components, such as SRP, soluble SR, or SecA/B, enabled synthesis of IMPs in a cell‐free manner by facilitating their integration into and translocation across the liposomal membranes.^11^ Therefore, mimicking the SRP/SR–translocon pathway is a promising approach for improving the fate of membrane proteins in cell‐free membrane protein synthesis/liposome systems.

**Figure 1:**
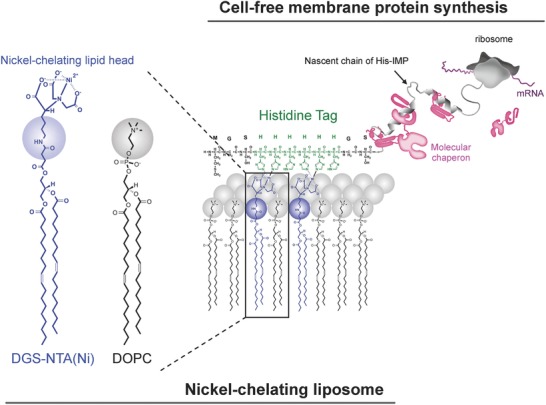
Schematic illustration of the His tag–fused membrane protein sorting system, via hexahistidine/nickel‐chelate affinity, using cell‐free membrane protein synthesis. Plasmid DNA encoding a N‐terminal hexahistidine (His) tag–fused membrane protein was constructed. NHis fused membrane proteins were synthesized using cell‐free protein synthesis in the presence of nickel‐chelating liposomes.

In this study, we developed an artificial membrane protein sorting system, constructed using polyhistidine/nickel‐chelate affinity and cell‐free membrane protein synthesis (**Figure** **1**). As proof of concept for this strategy, we selected the hexahistidine tag because this tag was one of the smallest tag and often used in the purification of many protein, and we assumed it would minimally affect the conformation of IMPs. We designed a N‐terminal hexahistidine‐fused conexin‐43 (NHis–Cx43) as a model membrane protein and prepared nickel‐chelating liposomes. Incorporation efficiency, oligomerization, and orientation of NHis–Cx43 in the liposomal membranes were investigated. Furthermore, the synergistic effects of a natural molecular chaperone, DnaK/DnaJ/GrpE (DnaKJE), in this artificial membrane protein sorting system were also examined, based on incorporation efficiency and NHis–Cx43 orientation. Finally, we demonstrated the versatility of this artificial membrane protein sorting system.

## 2 Results and Discussion

### 2.1 Liposome Sorting System Based on Nickel‐Chelate Affinity of a N‐Terminal Hexahistidine‐Fused Membrane Protein

We prepared nickel‐chelating liposomes using 1,2‐dioleoyl‐*sn*‐glycero‐3‐phosphatidylcholine (DOPC) liposomes containing various amounts of 1,2‐dioleoyl‐sn‐glycero‐3‐[(*N*‐(5‐amino‐1‐carboxypentyl) iminodiacetic acid) succinyl] (nickel salt) (DGS–NTA (Ni)). The average diameter of each liposome was about 110–120 nm by dynamic light scattering. The average zeta potential (ζ) of each liposome increased with DGS–NTA (Ni) concentrations. These values were −0.29, −12.7, −24.7, and −37.7 mV for DOPC liposomes of 2.5, 5.0, and 10 mol% DGS–NTA (Ni), respectively (Table S2, Supporting Information).

**Figure 2:**
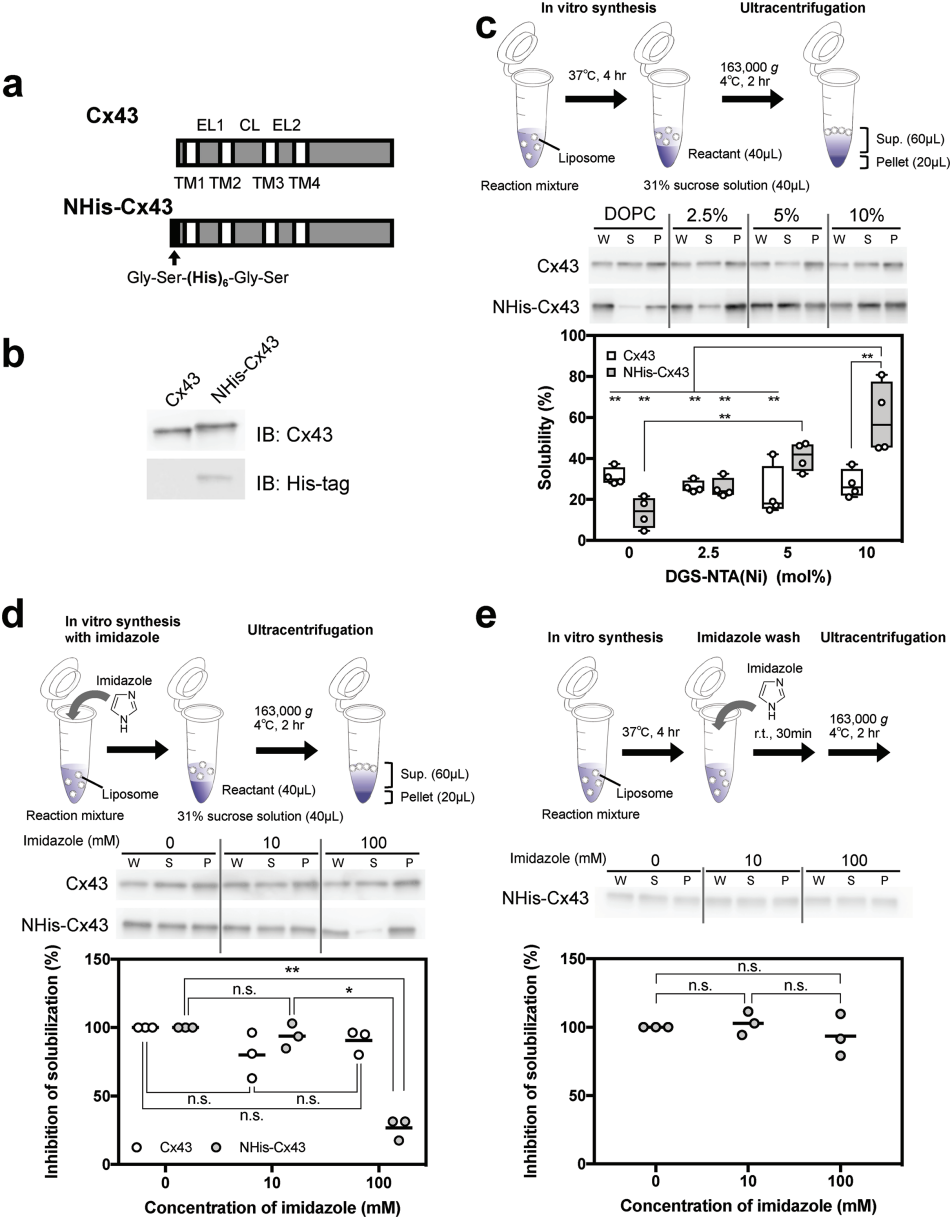
Efficient and specific solubilization of NHis–Cx43 via hexahistidine/nickel‐chelate affinity. a) Schematic illustration of Cx43 and NHis–Cx43 genes. The black arrow indicates the His tag (Gly–Ser–(His)_6_–Gly–Ser). EL, CL, and TM represent extracellular loop, cytoplasmic loop, and transmembrane, respectively. b) Western blot analysis of Cx43 and NHis–Cx43 synthesized by cell‐free protein synthesis in the absence of liposomes. Immunoblotting was performed using Cx43‐specific (top) or His‐tag‐specific (bottom) antibodies. c) Solubility of Cx43 and NHis–Cx43 in the presence of nickel‐chelating liposomes (0.5 × 10^−3^ m lipids), semiquantified by western blotting after ultracentrifugation. The expressed whole sample (W) was separated by ultracentrifugation, resulting in supernatant (S) and pellet (P). Each dot denotes an individual solubility (*n* = 4). Boxes span the interquartile range (25–75%); the line within each box denotes the median and whiskers indicate the minimum and maximum values. Two‐way ANOVA: significant effects of the hexahistidine tag (*F*_1, 24_ = 6.037, *p* = 0.0216), nickel‐chelating lipid concentration (*F*_3, 24_ = 8.304, *p* = 0.0006), and their interaction (*F*_3, 24_ = 11.19, *p* < 0.0001). **Adjusted *p* < 0.01, Tukey's post hoc test. d) Inhibition by imidazole of solubilization of synthesized Cx43 or NHis–Cx43, semiquantified by western blotting. Cell‐free synthesis of Cx43 or NHis–Cx43 was performed in the presence of 10 mol% nickel‐chelating liposomes (0.5 × 10^−3^ m lipids) and imidazole. Each dot denotes an individual inhibitory ratio (*n* = 3) and the line denotes the mean. One‐way factorial ANOVA: significant effects of imidazole on Cx43 synthesis (*F*_2,6_ = 2.49, *p* = 0.1632) and NHis–Cx43 synthesis (*F*_2,6_ = 101.7, *p* < 0.0001). *Adjusted *p* < 0.05, **adjusted *p* < 0.01, and n.s. (nonsignificant), Tukey's post hoc test. e) Detachment of nonintegrated NHis–Cx43 to liposomal surfaces with imidazole, semiquantified by western blotting. After cell‐free NHis–Cx43 synthesis in the presence of 10 mol% nickel‐chelating liposomes (0.5 × 10^−3^ m lipids), the product was incubated with various concentrations of imidazole. Each dot denotes an individual inhibitory ratio (*n* = 3), and the line denotes the mean. One‐way factorial ANOVA: significant effects of imidazole on NHis–Cx43 synthesis (*F*_2,6_ = 0.6647, *p* = 0.5486). n.s. (nonsignificant), Tukey's post hoc test.

Glycine–serine–hexahistidine–glycine–serine was genetically fused to the N‐terminus of Cx43 to obtain NHis–Cx43 (**Figure** **2**a), as confirmed by western blotting of the cell‐free synthesized protein (Figure 2b). Cell‐free synthesis of Cx43 or NHis–Cx43 was performed for 4 h in the absence of liposomes (Figure S1, Supporting Information). After ultracentrifugation, all synthesized Cx43 or NHis–Cx43 was detected in the pellet fraction, suggesting that both the protein preparations were aggregated. To evaluate effects of nickel‐chelating liposomes on protein solubilization, cell‐free synthesis of Cx43 or NHis–Cx43 was performed for 4 h in the presence of DOPC or DGS–NTA (Ni) liposomes at various mol% values (Figure 2c). Synthesized in this manner, Cx43 and NHis–Cx43 were detected in the supernatant fraction containing liposomes. The average solubility of Cx43 in each liposome was ≈30%, with little change among various liposomes containing DGS–NTA (Ni). In contrast, the solubility of NHis–Cx43 was higher with increased DGS–NTA (Ni) concentration, and was the highest in the presence of 10 mol% DGS–NTA (Ni) liposomes, designated as “nickel‐chelating liposomes.”

To further evaluate interaction of the hexahistidine of NHis–Cx43 with nickel‐chelating liposomes, an inhibition experiment was performed using imidazole (Figure 2d). Solubility of nontagged Cx43 synthesized in nickel‐chelating liposomes did not change in the presence of imidazole. In contrast, imidazole at 100 mmol L^−1^ significantly inhibited the chaperoning effect of the nickel‐chelating liposomes, that is, their promotion of protein solubilization. In another experiment, imidazole was added after completion of cell‐free NHis–Cx43 synthesis in nickel‐chelating liposomes. In this case, imidazole had no inhibitory effect (Figure 2e), indicating that synthesized NHis–Cx43 strongly interacted with the liposomes without chelating the Ni ions. Nickel‐chelating liposomes did not solubilize the aggregates formed during cell‐free synthesis of NHis–Cx43 without liposomes (Figure S2, Supporting Information). These results indicated that specific interactions between the hexahistidine in NHis–Cx43 and nickel‐chelating liposomes during protein synthesis were important for improving sorting efficiency of NHis–Cx43 into liposomes.

### 2.2 Hemichannel Formation of Newly Synthesized NHis–Cx43

**Figure 3:**
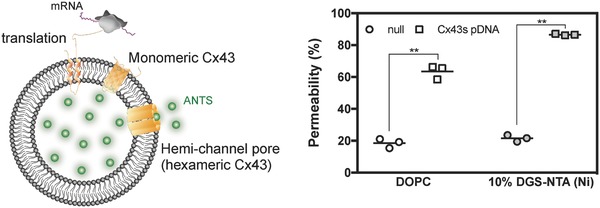
Synthesized NHis–Cx43 was integrated into liposomal membranes and formed a hemichannel pore of NHis–Cx43. Fluorescence intensity of Cx43/DOPC liposomes containing ANTS and NHis–Cx43/10 mol% nickel‐chelating liposomes, after addition of DPX. Each symbol denotes an individual data point showing ANTS permeability (*n* = 3) and the line denotes the mean. **p < 0.01, two‐tailed paired Welch's *t*‐test.

In biological systems, newly synthesized Cx43 molecules oligomerize and form hexameric hemichannels in the trans‐Golgi network^12^ and are subsequently translocated to the plasma membrane. In the plasma membrane, a pore formed by the Cx43 hemichannel enables passage of small molecules (molecular weight (MW) < 1200), maintaining environmental homeostasis between the cytoplasm and extracellular space.^13^ Therefore, it is important that a sorting system enables the monomeric Cx43 molecules, that when synthesized, are oligomerized to form hexameric hemichannels in the liposomal membranes. To confirm Cx43 hemichannel formation, we examined release of a fluorescent probe, 8‐aminonaphthalene 1,3,6‐trisulfonic acid (ANTS), from Cx43 containing liposomes.[[qv: 9c]] After cell‐free Cx43s synthesis in the presence of liposomes containing ANTS, any ANTS transferred to the outside of the liposomes was quenched with *p*‐xylene‐bis‐pyridinium bromide (DPX). **Figure** **3** clearly shows that ANTS permeated from the internal to the external aqueous phase of the liposomes in the presence of Cx43 or NHis–Cx43. Post‐translational modification of the C‐terminal of Cx43 regulates the biological activity and structure of the hexameric Cx43 hemichannel. In particular, Cx43 phosphorylation abolished the gate permeability of the hemichannel pore.^14^ As we previously discussed,[[qv: 9c]] PURESYSTEM is composed only of transcription/translation related factors with no phosphatases and the synthesized proteins are not phosphorylated. Consequently, the NHis–Cx43 hemichannel pore, when synthesized in a cell‐free system, maintains an open‐gate state in the liposomal membranes. These results suggested that NHis–Cx43 integrated through hexahistidine/nickel‐chelating affinity was laterally diffused into liposomal membranes, then subsequently oligomerized to form hexameric hemichannels similar to those formed in Cx43/DOPC proteoliposomes.

### 2.3 Topology of NHis–Cx43 Reconstituted into Liposomes

**Figure 4:**
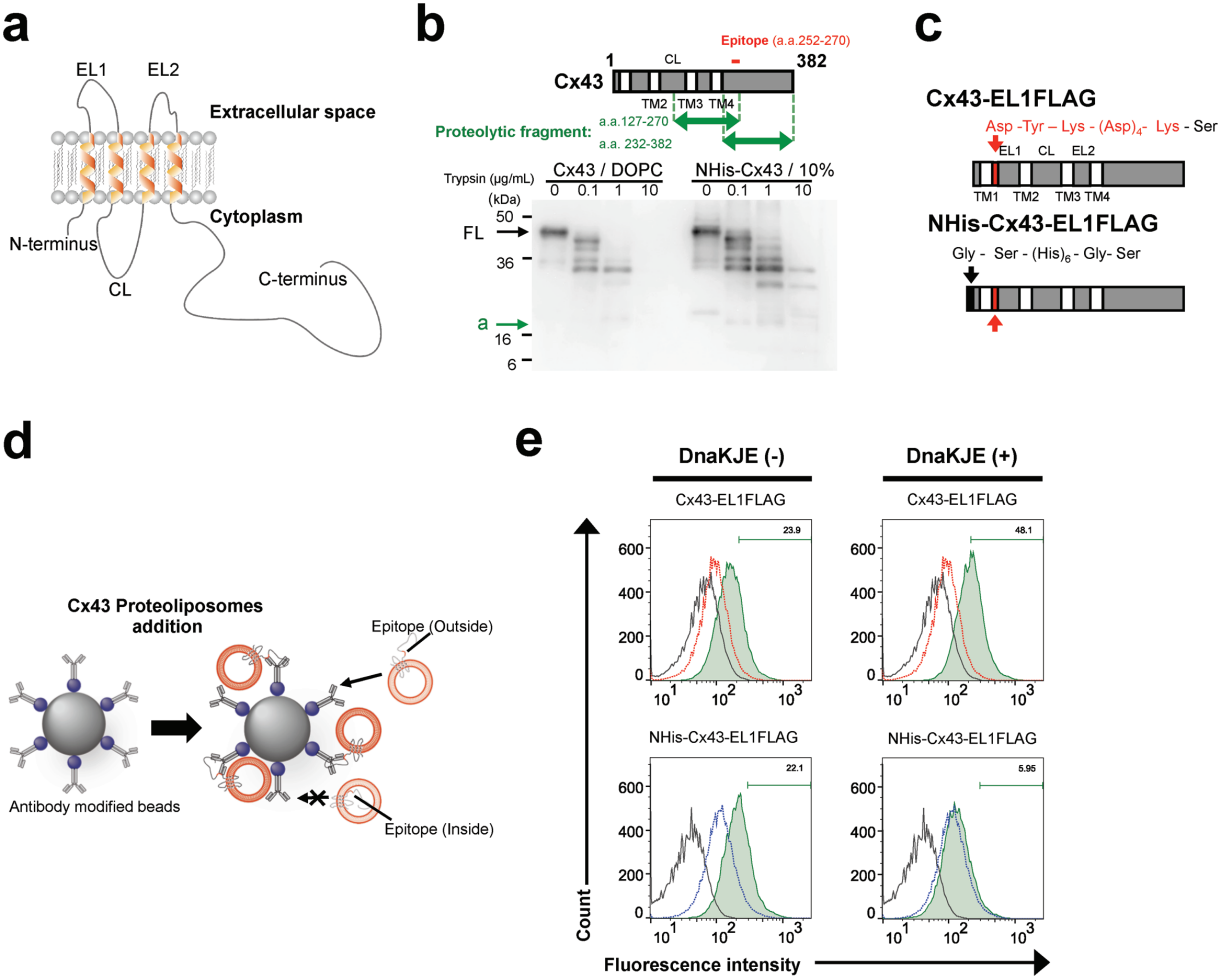
Orientation of NHis–Cx43 in the liposomal membrane. a) Schematic illustration of the orientation of Cx43 in living systems. The N‐terminus, CL, and C‐terminus of Cx43 are positioned at the cytoplasm. ELs protrude into the extracellular space. b) Schematic illustration of the detectable proteolytic fragments (top) and western blot analysis of proteolytic fragments of Cx43 and NHis–Cx43, using Cx43‐specific antibodies after trypsin treatment (bottom). The arrows indicate the full‐length Cx43 and NHis–Cx43 (FL) and a proteolytic fragment (a). Results are representative of three independent experiments. c) Schematic illustration of Cx43–EL1FLAG and NHis–Cx43–EL1FLAG fusion proteins. The black arrow indicates the His tag and the red arrow indicates the FLAG tag. d) Schematic illustration of flow cytometry following immunoprecipitation. e) Flow cytometry analysis of Cx43–EL1FLAG‐ or NHis–Cx43–EL1FLAG‐integrated proteoliposomes (green area), using a FLAG‐tag‐specific antibody. Solid lines indicate loading of FLAG‐tag‐specific antibody‐modified beads. Red and blue lines indicate loading of beads reacted with Cx43‐integrated proteoliposomes and NHis–Cx43‐integrated proteoliposomes, respectively.

The topology of integrated NHis–Cx43 in liposomal membranes was investigated by selective digestion of the membrane protein domains at the liposomal surface with proteases. Cx43 has four transmembrane domains, with the following orientations in living systems: cytoplasmic N‐, C‐terminal domains and the cytoplasmic loop are protruded into the cytoplasm and two extracellular loops are protruded into the extracellular space (**Figure** **4**a). The proteolytic fragments of NHis–Cx43 integrated into nickel‐chelating liposomes, with trypsin added outside of the liposomes, had similar electrophoretic mobilities to those of Cx43 in DOPC liposomes (Figure 4b).[[qv: 9c]] A band of ≈16.5 kDa (Figure 4b, arrow (a)) was derived from either the proteolytic fragment of the acral region of Cx43 between the fourth transmembrane and C‐terminal domains or from the cytoplasmic loop of Cx43. This was because the anti‐Cx43 antibody recognized the C‐terminal domain of Cx43 after the trypsin digestion (located at residues 252–270 of Cx43). Therefore, at least part of the integrated NHis–Cx43, the C‐terminal domain or cytoplasmic loop of the integrated NHis–Cx43 into the nickel‐chelating liposomal membranes, was protruding on the outside of the liposomes.

To further investigate the orientation of the integrated NHis–Cx43, we performed immunoprecipitation to detect epitopes of NHis–Cx43 on the outside of the liposomes. We designed and constructed four Cx43 epitope‐tagged fusion proteins (N‐terminal fusion: NFLAG–Cx43 and NHis, FLAG–Cx43 (Figure S3a, Supporting Information); extracellular Loop 1 fusion: Cx43–EL1FLAG and NHis–Cx43–EL1FLAG (Figure 4c)). We selected the FLAG tag (DYKDDDDK) because this epitope tag was hydrophilic and we assumed it would minimally affect the properties of the transmembrane domain of Cx43. Indeed, physicochemical simulation indicated that the predicted transmembrane domains of both NFLAG–Cx43 and NHis, FLAG–Cx43 were the same as those of the original Cx43 (Table S4, Supporting Information). In both Cx43–EL1FLAG and NHis–Cx43–EL1FLAG, the predicted amino acid sequences of the first transmembrane domains were slightly different from those of the original Cx43s (Table S4, Supporting Information).

Western blotting results on Cx43 constructs synthesized cell‐free in the absence of liposomes showed successfully construction of NFLAG–Cx43, Cx43–EL1FLAG, NHis, FLAG–Cx43, and NHis–Cx43–EL1FLAG (Figure S3b, Supporting Information). In addition, all synthesized Cx43 derivatives were also successfully solubilized in the presence of each liposome species in a manner similar to the corresponding original Cx43 or NHis–Cx43 proteins (Figure S3c, Supporting Information). Next, we performed the immunoprecipitation using antibody‐modified beads and qualitatively analyzed by flow cytometry (Figure 4d). Cx43, NFLAG–Cx43, Cx43–EL1FLAG, NHis–Cx43, NHis, FLAG–Cx43, and NHis–Cx43–EL1FLAG proteoliposomes were prepared, with all NHis–Cx43 derivatives synthesized cell‐free in the presence of nickel‐chelating/1,2‐dimyristoyl‐*sn*‐glycero‐3‐phosphorylethanolamine‐*N*‐(lissamine rhodamine B sulfonyl) (ammonium salt) (DMPE–RhoB) liposomes (liposomes modified with the fluorescent probe rhodamine B (RhoB)). After incubation of samples with an anti‐Cx43 or anti‐FLAG antibody, the fluorescence intensity of RhoB was measured by flow cytometry. For the anti‐Cx43 antibody binding to the C‐terminal domain of Cx43, every bead showed a positive signal (Figure S4, Supporting Information), consistent with the results of the protease protection assay (Figure 4b). The anti‐FLAG antibody‐modified beads showed positive signals after incubation with NFLAG–Cx43/nickel‐chelating/DMPE–RhoB proteoliposomes or NHis, FLAG–Cx43/nickel‐chelating/DMPE–RhoB proteoliposomes (Figure S5, Supporting Information). These results indicated that part of the N‐ and C‐terminal domains of the integrated Cx43 or NHis–Cx43 protruded outside the liposomes. At the initial phase of NHis–Cx43 synthesis in this system, the N‐terminal hexahistidine of nascent NHis–Cx43 would be anchored to the Ni–NTA on lipid headgroups located on the exterior surface of liposomal membranes. Therefore, the N‐terminal domain of NHis–Cx43 would be positioned on the outside of liposomes, anchored to Ni–NTA. Furthermore, anti‐FLAG antibody‐modified beads also showed positive signals after incubation with Cx43–EL1FLAG/nickel‐chelating/DMPE–RhoB proteoliposomes or NHis–Cx43–EL1FLAG/nickel‐chelating/DMPE–RhoB proteoliposomes (Figure 4e). This indicated that part of the extracellular loop 1 moiety in integrated Cx43 or NHis–Cx43 was also positioned on the outside of liposomes. These results suggested that the NHis–Cx43 integrated via hexahistidine/nickel‐chelating affinity showed a dual topology in liposomal membranes.

### 2.4 Molecular Chaperone Regulated Topology of NHis–Cx43

We investigated the effects of a natural molecular chaperone on the topology of NHis–Cx43 to reconstitution to nickel‐chelating liposomes in cell‐free membrane protein synthesis (Figure 4e and Figures S4 and S5 (Supporting Information)). The molecular chaperone DnaKJE binds to the hydrophobic nascent polypeptide^15^ with formation of the ribosome–nascent chain/DnaKJE complex, preventing aggregation of nascent polypeptides during cell‐free protein synthesis.^16^ Addition of DnaKJE resulted in increased solubility of all Cx43–FLAG fusion proteins synthesized in cell‐free synthesis, indicating that DnaKJE acted as a molecular chaperone for reconstitution of Cx43–FLAG to liposomes (Figure S6, Supporting Information). After incubation with anti‐Cx43 antibody‐modified beads, every bead showed positive signals and the signals tended to be stronger in the presence of DnaKJE (Figure S4, Supporting Information). NFLAG–Cx43/nickel‐chelating /DMPE–RhoB proteoliposomes, NHis, FLAG–Cx43/nickel‐chelating/DMPE–RhoB proteoliposomes, and Cx43–EL1FLAG/nickel‐chelating/DMPE–RhoB proteoliposomes also showed positive signals in the presence of DnaKJE after incubation with anti‐FLAG antibody‐modified beads (Figure S5 (Supporting Information) and Figure 4e). In Cx43–EL1FLAG/nickel‐chelating/DMPE–RhoB proteoliposomes, the positive signals were increased from 23.9% to 48.1% in the presence of DnaKJE. In contrast, we noted that the signal by anti‐FLAG antibody in only the NHis–Cx43–EL1FLAG/nickel‐chelating system was not detected in the presence of DnaKJE, the positive signals were decreased from 22.1% to only 5.95% (Figure 4e). Almost all extracellular loop 1 domains of NHis–Cx43 should be positioned on the inside of the liposomes. DnaKJE prevented aggregation of a wide range of proteins^16^, ^17^ and also contributed to the membrane insertion of the C‐tailed anchored protein, TssL, into the inner membrane of *E. coli*.^18^ Therefore, a plausible explanation for our findings was that DnaKJE was bound to the nascent polypeptide chain anchored to the Ni–NTA moieties on the lipid headgroup, preventing their hydrophobicity‐driven random insertion. In addition, the bigger hydrophilic C‐terminal domain of Cx43 probably was unable to penetrate into the bilayer membrane and localize to the outer surface of the liposomes. Together, these findings suggested that natural molecular chaperones controlled the orientation of NHis–Cx43 inserted into liposomes only in the nickel‐chelating sorting systems and that proteoliposomes with single topology were constructed.

### 2.5 Application to Other Membrane Proteins, Including β‐Barrel Membrane Proteins

Finally, to examine the versatility of this membrane protein sorting system, we selected three membrane proteins; *Streptomyces lividans* potassium channel KcsA, *E. coli* hydrogenase‐1 small chain HyaA, and *E. coli* outer membrane protein A OmpA. HyaA was selected as an aggregation‐prone model membrane protein that was one of the three least solubilized membrane proteins in the cell‐free membrane protein synthesis/liposome system, as previously reported.[[qv: 9f]] OmpA was selected as a β‐barrel membrane protein.^19^ KcsA, HyaA, and OmpA also had glycine–serine–hexahistidine–glycine–serine groups genetically fused to the N‐terminus of each membrane protein to produce NHis–KcsA, NHis–HyaA, and NHis–OmpA, respectively.

**Figure 5:**
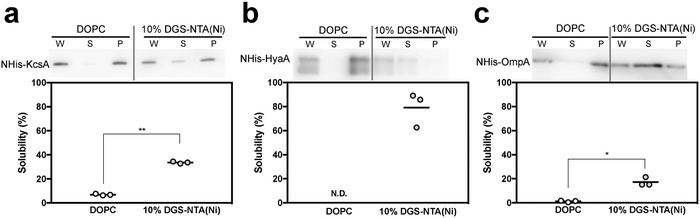
Versatility of the NHis–membrane protein sorting system utilizing polyhistidine/nickel‐chelate affinity. Western blot analysis of synthesized a) His–KcsA, b) His–HyaA, and c) His–OmpA, using cell‐free protein synthesis in the presence of DOPC or 10 mol% nickel‐chelating liposomes. Each dot denotes an individual solubility (*n* = 3), and the line denotes the mean. *p < 0.05 and **p < 0.01, two‐tailed Welch's *t*‐test. N.D., not detected.

Cell‐free membrane protein synthesis was performed in the absence of liposomes or in the presence of DOPC liposomes or nickel‐chelating liposomes and the solubility of each membrane protein was semiquantified by western blotting using an anti‐His‐tag antibody (**Figure** **5**). In the absence of liposomes, most of the three membrane proteins were detected in the pellets after ultracentrifugation (Figure S7, Supporting Information). NHis–KcsA and NHis–OmpA were slightly solubilized in the presence of DOPC liposomes (Figure 5a,c). Almost all the NHis–HyaA was aggregated (Figure 5b). In the presence of nickel‐chelating liposomes, the amounts of NHis–KcsA, NHis–HyaA, and NHis–OmpA in the supernatant fraction were significantly increased from 6.87 ± 0.69% to 33.6 ± 0.86% for NHis–KcsA, from almost 0% to 87.5 ± 14.4% for NHis–HyaA, and from 1.20 ± 0.69% to 17.2 ± 3.6% for NHis–OmpA. These results indicated that the polyhistidine/nickel‐chelate affinity‐based membrane protein sorting system was an efficient preparation method of proteoliposomes integrated with not only α‐helix but also β‐barrel membrane proteins. Several studies demonstrated that translocon‐reconstituted proteoliposomes were useful for improved integration efficiency and orientation of IMPs under cell‐free membrane protein synthesis.^11^ Furthermore, membrane structure, such as surface charge and lipid composition, also affected the membrane protein topogenesis.^20^ Therefore, a combination of our system and other approaches could result in further improved proteoliposome preparation methods.

## 3 Conclusion

We demonstrated a simple membrane protein sorting system, based on cell‐free N‐terminal hexahistidine‐fused membrane protein synthesis in the presence of nickel‐chelating liposomes. In our system, the nascent polypeptide of NHis–membrane proteins interacted with the nickel‐chelating lipid head on liposomes, subsequently integrating into liposomal membranes and forming functional oligomers. A combination of this present system and molecular chaperones would be capable of regulating the orientation of integrated NHis–membrane proteins. Furthermore, this system was also applied to prepare both α‐helix and β‐barrel membrane protein–integrated proteoliposomes efficiently. Taken together, our results suggested that a membrane protein sorting system utilizing the polyhistidine/nickel‐chelate affinity and molecular chaperones is a promising method to prepare proteoliposomes. This should accelerate elucidation of biological phenomena, development of new platforms for biosensing, and discovery of drug delivery nanocarrier‐based systems.

## 4 Experimental Section

*Plasmid DNA Construction*: The rat Cx43‐expressing plasmid DNAs (pDNAs) pURE–Cx43 were constructed as described previously,[[qv: 9c]] denoted then as “pURE2–Cx43.” To prepare polymerase chain reaction (PCR)–amplified DNA encoding Cx43s, PCR products were amplified using the primers listed in Table S1 (Supporting Information). The pURE–NHis–Cx43 plasmid was constructed by inserting the *NcoI*/*SmaI* PCR‐amplified complementary DNA (cDNA) fragments into the *NcoI*/*SmaI* site of the pURE2 vector (Cosmo Bio, Tokyo, Japan). The pURE–Cx43–EL1FLAG and pURE–NHis–Cx43–EL1FLAG plasmids were constructed by inserting the annealed oligonucleotides listed in Table S1 (Supporting Information) into the *PvuII* site of extracellular loops (EL) 1 domain of the pURE–Cx43 and pURE–NHis–Cx43 sequences, respectively. N‐terminal hexahistidine‐fused *E. coli* hydrogenase‐1 small chain, NHis–HyaA and outer membrane protein A, NHis–OmpA, cDNAs without signal peptide sequences were synthesized by Eurofins Genomics (Ebersberg, Germany). The pURE–NHis–HyaA and pURE–NHis–OmpA plasmids were constructed by inserting the *NdeI*/*HindIII* NHis–HyaA or NHis–OmpA cDNA fragment into the *NdeI*/*HindIII* site of the pURE1 vector (Cosmo Bio). All pDNAs were amplified in the DH5α strain of *E. coli*. The plasmid DNA was then purified using a PureLink HiPure Plasmid Midiprep Kit (Thermo Fisher Scientific, Waltham, MA, USA).

*Liposome Preparation*: DOPC, DGS–NTA (Ni), and DMPE–RhoB were purchased from Avanti Polar Lipids (Alabaster, AL, USA). Liposomes were prepared using the conventional extrusion method.[[qv: 9d]] The following lipid mixtures were used: DOPC, [DOPC/DGS–NTA (Ni) (97.5:2.5, 95:5, 90:10) mol% ratio] and [DOPC/DGS–NTA (Ni)/DMPE–RhoB (180:20:1)]. The lipids were dissolved in methanol/chloroform (1/1, v/v) and each sample was placed in a glass micro‐test tube and gently evaporated by rotation under flowing argon gas. The lipid film, dried overnight, was hydrated with 50 × 10^−3^ m 4‐(2‐hydroxyethyl)‐1‐piperazineethanesulfonic acid (HEPES) buffer (pH 7.5), incubated for 2 h at 37 °C, and vortexed lightly for 30 s to suspend all the materials in the tube. Following hydration, liposome suspensions were extruded with a mini‐extruder (Avanti Polar Lipids) equipped with a 0.1 µm pore polycarbonate membrane (Whatman, Maidstone, UK) at 50 °C. The average size and zeta (ζ) potential of liposomes, large unilamellar vesicles, were measured using a Zetasizer Nano ZSP instrument (Malvern Instrument Inc., Worcestershire, UK) and the results are summarized in Table S2 (Supporting Information). The lipid concentration was measured using the Phospholipid C‐Test (Wako, Osaka, Japan).

*Preparation and Purification of Membrane Protein–Integrated Proteoliposomes*: In vitro protein synthesis of membrane proteins was performed using PURESYSTEM (PUREfrex1.0; GeneFrontier, Chiba, Japan) according to the manufacturer's instructions. Reaction mixtures, each containing 4 ng µL^−1^ pDNA, were prepared with or without liposomes at a final concentration of 0.5 × 10^−3^ m lipids.[[qv: 9d]] These were incubated without agitation for 4 h at 37 °C in a heat block incubator. For purification, 40 µL aliquots of each sample were overlaid with 40 µL 31% w/v sucrose solution and ultracentrifuged at 163 000 × *g*, 4 °C for 2 h. The 60 µL upper layer was collected and designated as the supernatant sample and the lower 20 µL fraction was collected and designated as the pellet sample.

*Western Blot Analysis*: Samples were separated by sodium dodecyl sulfate polyacrylamide gel electrophoresis (SDS‐PAGE) under reducing conditions and bands transferred electrophoretically to a polyvinylidene difluoride (PVDF) membrane. The PVDF membrane was loaded onto a SNAP i.d. 2.0 Protein Detection System (Millipore, Billerica, MA, USA) and incubated with Blockingone (Nacali Tesque, Kyoto, Japan). To detect Cx43 (C‐terminal domain of Cx43), the membrane was reacted with a mouse anti‐connexin‐43 monoclonal IgG (1:1000 dilution; BD Transduction Laboratories, Lexington, KY, USA) for 10 min at room temperature and subsequently incubated for 10 min at room temperature with goat anti‐mouse IgG conjugated with horseradish peroxidase (1:3000 dilution; Santa Cruz Biotechnology, Santa Cruz, CA, USA.[[qv: 9c]] To detect hexahistidine, the membrane was similarly incubated with a mouse anti‐His_6_ monoclonal IgG (1:2000 dilution; Roche Diagnostics, Mannheim, Germany) followed by goat anti‐mouse IgG conjugated with horseradish peroxidase (1:4000 dilution; Santa Cruz Biotechnology). The membranes were each stained with ECL Western Blotting Detection Reagents (GE Healthcare, Milwaukee, MI, USA) and bands were visualized using an LAS‐4000 EPUV mini (FUJIFILM, Tokyo, Japan). Chemiluminescence intensities were measured at appropriate sample dilutions, as listed in Table S3 (Supporting Information). Semiquantitative analysis of solubility (%) was performed using the following equation

(1) $$\mathrm{Solubility}\left(\%\right)=100\times \frac{\left(Int.\;\mathrm{of}\;S\right)\times 60}{\left(Int.\;\mathrm{of}\;S\right)\times 60+\left(Int.\;\mathrm{of}\;P\right)\times 20}$$

where *Int. of S* and *Int. of P* are the chemiluminescence intensity per 1 µL sample, for supernatant and pellet, respectively.

*Inhibition of Hexahistidine/Nickel‐Chelate Affinity by Imidazole*: To confirm the effects of hexahistidine/nickel‐chelate affinity on solubilization of membrane proteins, in vitro membrane protein synthesis was performed in the presence of both 10 mol% DGS–NTA (Ni) liposomes (0.5 × 10^−3^ m lipids) and imidazole (Wako) at the indicated concentrations. Separately, following cell‐free synthesis of NHis–Cx43 in the presence of 10 mol% DGS–NTA (Ni) liposomes (0.5 × 10^−3^ m lipids), the reactant was incubated with imidazole at the indicated concentrations for 30 min at room temperature. Purification was performed as described under subsection “Preparation and Purification of Membrane Protein–Integrated Proteoliposomes” and proteins were analyzed by western blotting.

*Dye Transfer Assay*: A hemichannel‐formed Cx43‐dependent dye transfer assay was conducted as described previously.[[qv: 9c]] Briefly, cell‐free Cx43s protein synthesis was performed for 4 h in the presence of liposomes (0.5 × 10^−3^ m lipid), using liposomes that had been hydrated with 50 × 10^−3^ m HEPES buffer (pH 7.5) containing 25 × 10^−3^ m ANTS (Molecular Probes/Thermo Fisher Scientific, Eugene, OR, USA). Immediately after cell‐free Cx43s synthesis, reactants were mixed with an equal volume of 50 × 10^−3^ m HEPES buffer (pH 7.5), with or without 1% Triton X‐100. Subsequently, each sample was mixed with a half volume of 50 × 10^−3^ m HEPES buffer containing 90 × 10^−3^ m DPX (Molecular Probes/Thermo Fisher Scientific). Fluorescence intensities were measured with a fluorescence spectrometer (FP‐8500; JASCO, Tokyo, Japan) at an excitation wavelength of 350 nm and emission wavelength of 530 nm.

*Analysis of Proteolytic Fragments of Cx43*: Supernatant samples containing Cx43‐integrated DOPC liposomes or NHis–Cx43‐integrated 10 mol% DGS–NTA (Ni) liposomes were incubated with the indicated concentrations of sequencing‐grade modified trypsin (Promega, Madison, WI, USA) for 30 min at 37 °C. After the incubation, proteolytic fragments of Cx43 were evaluated by western blotting.

*Flow Cytometry Analysis of the Topology of Integrated Cx43 Derivatives*: Preparation of proteoliposomes integrated with Cx43 derivatives was performed with or without molecular chaperones containing DnaK, DnaJ, and GrpE (DnaK mix; GeneFrontier) as described under subsection “Preparation and Purification of Membrane Protein‐Integrated Proteoliposomes.” PureProteome Protein G magnetic beads (Millipore) (2 × 10^5^ particles per tube) were incubated with 20 µg mL^−1^ mouse anti‐FLAG M2 IgG (Sigma‐Aldrich, St. Louis, MO, USA) with rotation for 1 h at room temperature. After incubation, beads were washed three times with phosphate buffered saline with 0.02% Tween 20 (PBSt) on a magnetic stand and then blocked with assay diluent (Affymetrix/eBioscience, San Diego, CA, USA)) containing 1 × 10^−3^ m DOPC (liposomes) for 1 h at room temperature. Following blocking, beads were washed three times with PBSt and then incubated at 4 °C overnight with DOPC/DGS–NTA (Ni)/Rho–DMPE liposomes integrated with Cx43 derivatives (total lipid concentration, 0.05 × 10^−3^ m) in assay diluent containing 0.1 × 10^−3^ m DOPC and 100 × 10^−3^ m imidazole. The RhoB fluorescence signals were measured by flow cytometry (LSR Fortessa cell analyzer; BD Bioscience, San Jose, CA, USA). The total fluorescence intensities of samples containing 3 × 10^4^ beads were measured and analyzed with FlowJo software (Treestar, Inc., San Carlos, CA, USA). The gate was drawn to include at least 80% of detected events. More than cut‐off value calculated as the mean of Cx43 or NHis–Cx43 groups + 2 standard deviation (SD) was determined as the positive signals.

*Statistical Analysis*: Differences in solubilities of Cx43 and NHis–Cx43 in the presence of various liposomes were assessed using two‐way factor analysis of variance (ANOVA) followed by Tukey's multiple comparisons test. Inhibition by imidazole of Cx43 and NHis–Cx43 solubility was assessed using one‐way factorial ANOVA followed by Tukey's multiple comparisons' test. Differences in dye transfer assay results were assessed with two‐tailed Welch's *t*‐test. Differences in solubilities of His–KcsA and His–OmpA were also evaluated with two‐tailed Welch's *t*‐test. Adjusted *p* < 0.05 and *p* < 0.05 values were considered to be statistically significant. All statistical analyses were performed using Graphpad Prism 7 (GraphPad Software, Inc., La Jolla, CA, USA).

## Conflict of Interest

The authors declare no conflict of interest.

## Supporting information

Supplementary
