# Supplementary material for: Proteoliposome Engineering with Cell‐Free Membrane Protein Synthesis: Control of Membrane Protein Sorting into Liposomes by Chaperoning Systems
Source: Adv Sci (Weinh). 2018 Aug 23;5(10):1800524. doi: 10.1002/advs.201800524 (PMC6193158; doi:10.1002/advs.201800524)
Supplement: Supplementary file 1 — Supplementary [file ADVS-5-1800524-s001.pdf]

Copyright WILEY-VCH Verlag GmbH & Co. KGaA, 69469 Weinheim, Germany, 2018.

ADVANCED SCIENCE
Open Access

# Supporting Information

for *Adv. Sci.,* DOI: 10.1002/advs.201800524

# Proteoliposome Engineering with Cell-Free Membrane Protein Synthesis: Control of Membrane Protein Sorting into Liposomes by Chaperoning Systems

*Mitsuru Ando, Shun Schikula, Yoshihiro Sasaki, and Kazunari Akiyoshi**

# Supporting Information
# for

## Proteoliposome Engineering with Cell-free Membrane Protein Synthesis: Control of Membrane Protein Sorting into Liposomes by Chaperoning Systems

Mitsuru Ando[1, 2], Shun Schikula[1], Yoshihiro Sasaki[1] and Kazunari Akiyoshi*[1,2]

1. Department of Polymer Chemistry, Graduate School of Engineering, Kyoto University, Katsura, Nishikyo-ku, Kyoto, 615-8510, Japan.

2. Japan Science and Technology Agency (JST), The Exploratory Research for Advanced Technology (ERATO), Bio-nanotransporter Project, Katsura Int'tech Center, Katsura, Nishikyo-ku, Kyoto 615-8530, Japan.

*: corresponding author

**Materials and Methods**

Plasmid DNA construction

N-terminal hexahistidine fused *Streptomyces lividans* potassium channel, KcsA, -expressing plasmid DNAs (pDNA) pURE-NHis-KcsA were constructed as described previously, denoted then as pURE-His6-KcsA[S1]. To prepare the polymerase chain reaction (PCR)- amplified DNA encoding Cx43s, PCR products were amplified by using the primers listed in Table S1. pURE-NFLAG-Cx43 and pURE-NHis, FLAG-Cx43 were constructed by inserting the *NcoI*/*SmaI* PCR-amplified cDNA fragments into the *NcoI*/*SmaI* site of the pURE2 vector (Cosmo Bio, Tokyo, Japan). All the pDNAs were amplified in the DH5α strain of *E. coli.* The plasmid DNA was purified using a PureLink® HiPure Plasmid Midiprep Kit (Thermo Fisher Scientific, Waltham, MA, USA).

Preparation and purification of membrane protein-integrated proteoliposomes

The in vitro protein synthesis of membrane proteins was performed using PURESYSTEM (PUREfrex®1.0; GeneFrontier, Chiba, Japan) according to the manufacturer's instructions. Reaction mixtures, each containing 4 ng/µL pDNA, was prepared with or without liposomes at a final concentration of 0.5 mM lipids. These were incubated without agitation for 4 h at 37°C in a heat block incubator. For purification, 40 µL aliquots of each samples were overlaid with 40 µL of 31% (w/v) sucrose solution and ultracentrifuged at 163,000 × g, 4°C for 2 h. The 60 µL upper layer was collected and designed as the supernatant sample and the lower 20 µL fraction was collected and designed as the pellet sample.

Western blot analysis

Samples were separated by sodium dodecyl sulfate-polyacrylamide gel electrophoresis (SDS-PAGE) under reducing conditions and bands transferred electrophoretically to a polyvinylidene difluoride (PVDF) membrane. The PVDF membrane was loaded onto a SNAP i.d. ® 2.0 Protein Detection System (Millipore, Billerica, MA, USA), and incubated with Blockingone (Nacali Tesque, Kyoto, Japan). To detect the Cx43 (C-terminal domain of Cx43), the membrane was reacted with a mouse anti-connexin-43 monoclonal IgG (1: 1000 dilution; BD Transduction Laboratories™, Lexington, KY, USA) for 10 min at room temperature and subsequently incubated for 10 min at room temperature with goat anti-mouse IgG conjugated with horseradish peroxidase (1:3000 dilution; Santa Cruz Biotechnology, Santa

Cruz, CA, USA). To detect hexahisdine, the membrane was reacted with a mouse anti-$His_6$ monoclonal IgG (1:2000 dilution; Roche Diagnostics, Mannheim, Germany), and subsequently reacted with goat anti-mouse IgG conjugated with horseradish peroxidase (1:4000 dilution; Santa Cruz Biotechnology). To detect FLAG, the membrane was reacted with mouse anti-FLAG® M2 IgG (1:1000 dilution; Sigma-Aldrich, St. Louis, MO, USA), and subsequently reacted with goat anti-mouse IgG conjugated with horseradish peroxidase (1: 2000 dilution; Santa Cruz Biotechnology) as described above. The membranes were each stained with ECL Western Blotting Detection Reagents (GE Healthcare, Milwaukee, MI, USA) and bands were visualized using an LAS-4000 EPUV mini (FUJIFILM, Tokyo, Japan).

Interaction of the post synthesized NHis-Cx43 and nickel-chelating liposome

To confirm the interaction of the post synthesized NHis-Cx43 with 10 mol% DGS-NTA (Ni) liposomes, the cell-free NHis-Cx43 synthesis was performed in the absence of any liposomes. After the synthesis, 10 mol% DGS-NTA (Ni) liposomes (0.5 mM lipids) was added and incubated at 4°C over night. The purification was performed as described above, and evaluated by western blotting.

Flow cytometry analysis of the topology of integrated-Cx43 derivatives

Preparation of proteoliposomes integrated with Cx43-derivatives were performed with or without the presence of molecular chaperones containing DnaK, DnaJ and GrpE (DnaK mix; GeneFrontier). PureProteome™ Protein G magnetic beads (Millipore) ($2 \times 10^5$ particles/tube) were incubated with 20 µg/mL mouse anti-connexin-43 monoclonal IgG (BD Transduction Laboratories™) or mouse anti-FLAG® M2 IgG (Sigma-Aldrich) with rotation for 1h at room temperature. After incubation, beads were washed three times with phosphate buffered saline with 0.02% Tween 20 (PBSt) on a magnetic stand and then blocked with assay diluent (Affymetrix/eBioscience, San Diego, CA, USA)) containing 1mM DOPC (liposomes) for 1 h at room temperature. Following blocking, beads were washed three times with PBSt and then incubated at 4°C overnight with DOPC/DGS-NTA(Ni)/ Rho-DMPE liposomes integrated with Cx43 derivatives (total lipid concentration was 0.05 mM) in assay diluent containing 0.1 mM DOPC and 100 mM imidazole. The rhodamine (RhoB) fluorescence signals were measured by using flow cytometery (LSR Fortessa cell analyzer; BD Bioscience, San Jose, CA, USA).

The total fluorescence intensities of samples containing $3 \times 10^4$ beads were measured and analyzed with FlowJo software (Treestar, Inc., San Carlos, CA, USA). The gate was drawn to include at least 80% of detected events.

**Table S1. Sequence of the oligonucleotides used in this study**

| Name | Sequence |
|---|---|
| NHis-Cx43 forward | 5’-CATCCATGGGATCGCATCACCATCACCATCACGGATCAGGTGACTGGAGT-3’ |
| NHis, FLAG-Cx43 forward | 5’-CATCCATGGGATCGCATCACCATCACCATCACGGATCAGACTACAAGGACGAT GACGACAAGGGTGACTGGAGT-3’ |
| NFLAG-Cx43 forward | 5’-CATCCATGGACTACAAGGACGATGACGACAAGGGTGACTGGAGT-3’ |
| Cx43 reverse | 5’-AACCCGGGTTAAATCTCCAGGTCATC-3’ |
| EL1FLAG sense | 5’-ACTACAAGGACGATGACGACAAGTCAG-3’ |
| EL1FLAG antisense | 5’-CTGACTTGTCGTCATCGTCCTTGTAGT-3’ |

**Table S2. Size and zeta potential of liposomes.** Results are expressed as means ± standard deviations (n = 3).

| | **Size (d.nm)** | **PdI** | **Zeta-potential (mV)** |
|---|---|---|---|
| DOPC | 119.7 ± 0.53 | 0.054 | -0.29 ± 0.17 |
| DOPC/DGS-NTA(Ni) (97.5/2.5) | 116.8 ± 0.53 | 0.081 | -12.7 ± 0.80 |
| DOPC/DGS-NTA(Ni) (95/5) | 110.4 ± 1.04 | 0.063 | -24.7 ± 0.93 |
| DOPC/DGS-NTA(Ni) (90/10) | 111.1 ± 1.17 | 0.057 | -37.7 ± 1.66 |
| DOPC/DGS-NTA(Ni)/DMPE-RhoB (180/20/1) | 111.6 ± 0.40 | 0.078 | -41.7 ± 1.97 |

**Table S3. Sample dilution for western blot analysis.**

| | DGS-NTA (Ni) conc. (mol%) | Whole | Supernatant | Pellet |
|---|---|---|---|---|
| **Cx43** | 0 | 1:40 | 1:6 | 1:40 |
| | 2.5 | | | |
| | 5 | | | |
| | 10 | | | |
| **NHis-Cx43** | 0 | 1:80 | 1:12 | 1:80 |
| | 2.5 | | 1:12 | |
| | 5 | | 1:24 | |
| | 10 | | 1:24 | |
| **NFLAG-Cx43** | 10 | 1:40 | 1:6 | 1:40 |
| **NHis, FLAG-Cx43** | 10 | 1:80 | 1:24 | 1:80 |
| **Cx43-EL1FLAG** | 10 | 1:40 | 1:6 | 1:40 |
| **NHis-Cx43-EL1FLAG** | 10 | 1:80 | 1:24 | 1:80 |
| **NHis-KcsA** | 0 | 1:80 | 1:40 | 1:80 |
| | 10 | | | |
| **NHis-HyaA** | 0 | 1:40 | 1:4 | 1:40 |
| | 10 | | 1:20 | |
| **NHis-OmpA** | 0 | 1:80 | 1:2 | 1:80 |
| | 10 | | | |

**Table S4. Transmembrane domain of Cx43 derivatives**

| | | Position | Amino Acid sequence |
|---|---|---|---|
| Cx43 | TM1 | 22 - 44 | *GKVWLSVLFIFRILLLG*TAVES |
| | TM2 | 72 - 94 | ISHVRFWVLQIIFVSVPTLLYLA |
| | TM3 | 156 - 178 | IISILFKSVFEVAFLLIQWYIYG |
| | TM4 | 208 - 230 | IFIIFMLVVSLVSLALNIIELFY |
| NFLAG-Cx43 | TM1 | 30 - 52 | *GKVWLSVLFIFRILLLG*TAVESA |
| | TM2 | 80 - 102 | ISHVRFWVLQIIFVSVPTLLYLA |
| | TM3 | 164 - 186 | IISILFKSVFEVAFLLIQWYIYG |
| | TM4 | 216 - 238 | IFIIFMLVVSLVSLALNIIELFY |
| Cx43-EL1FLAG | TM1 | 17 - 38 | YSTAG*GKVWLSVLFIFRILLLG* |
| | TM2 | 81 - 103 | ISHVRFWVLQIIFVSVPTLLYLA |
| | TM3 | 165 - 187 | IISILFKSVFEVAFLLIQWYIYG |
| | TM4 | 217 - 239 | IFIIFMLVVSLVSLALNIIELFY |
| | | | |
| NHis-Cx43 | TM1 | 32 - 54 | *GKVWLSVLFIFRILLLG*TAVESA |
| | TM2 | 82 - 104 | ISHVRFWVLQIIFVSVPTLLYLA |
| | TM3 | 166 - 188 | IISILFKSVFEVAFLLIQWYIYG |
| | TM4 | 218 - 240 | IFIIFMLVVSLVSLALNIIELFY |
| NHis, FLAG-Cx43 | TM1 | 40 - 62 | *GKVWLSVLFIFRILLLG*TAVESA |
| | TM2 | 90 - 112 | ISHVRFWVLQIIFVSVPTLLYLA |
| | TM3 | 174 - 196 | IISILFKSVFEVAFLLIQWYIYG |
| | TM4 | 226 - 248 | IFIIFMLVVSLVSLALNIIELFY |
| NHis-Cx43-EL1FLAG | TM1 | 27 - 48 | YSTAG*GKVWLSVLFIFRILLLG* |
| | TM2 | 91 - 113 | ISHVRFWVLQIIFVSVPTLLYLA |
| | TM3 | 175 - 197 | IISILFKSVFEVAFLLIQWYIYG |
| | TM4 | 227 - 249 | IFIIFMLVVSLVSLALNIIELFY |

The italic letters represent same sequences of TM1.

Locations of transmembrane domain of Cx43 derivatives and NHis-Cx43 derivatives were calculated using *SOSUI WWW server ver. 1.11*[S2]. Available at: http://harrier.nagahama-i-bio.ac.jp/sosui/.

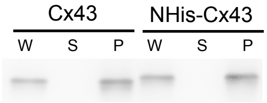


**Figure S1. Aggregation-prone Cx43 and NHis-Cx43 synthesized without liposomes.** Western blot analysis of Cx43 and NHis-Cx43 synthesized using cell-free protein synthesis in the absence of liposomes. The expressed whole sample (W) was ultracentrifugated, and collected supernatants (S) and pellets (P).

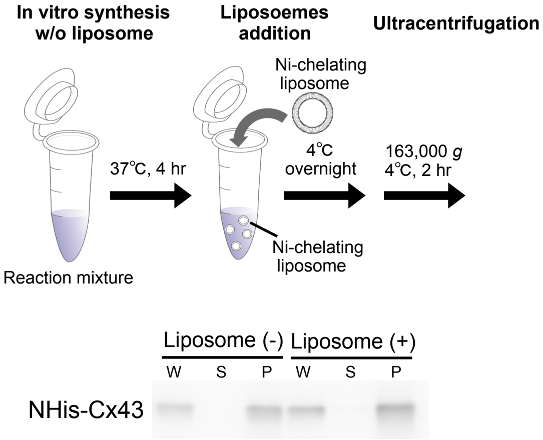


**Figure S2. Incubation of non-integrated NHis-Cx43 synthesized with nickel-chelating liposomes.** After cell-free NHis-Cx43 synthesis in the absence of liposomes, the reactant was incubated with 10 mol% nickel-chelating liposomes at 4°C overnight. The expressed whole sample (W) was ultracentrifugated, and collected supernatants (S) and pellets (P).

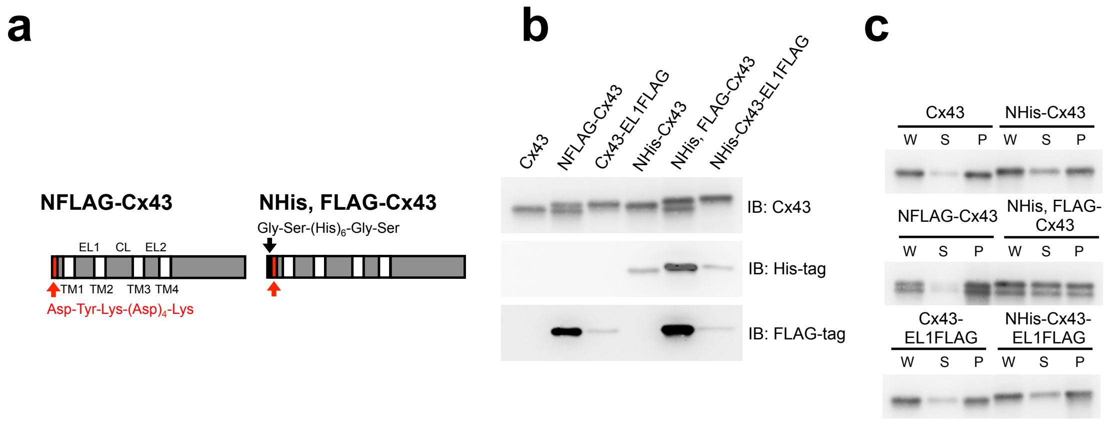


**Figure S3. Construction of Cx43-FLAG fusion protein.** (a) Schematic illustration of NFLAG-Cx43 and NHis, FLAG-Cx43 fusion proteins. Black arrow indicates His-tag and red arrow indicates FLAG-tag. **(b)** Western blot analysis of Cx43 derivatives and NHis-Cx43 derivatives synthesized by cell-free protein synthesis in the absence of any liposomes. Immunoblots were performed by using Cx43-specific antibody (top), His-tag-specific antibody (middle) and FLAG-tag-specific antibody (bottom). **(c)** Western blot analysis of Cx43 derivatives and NHis-Cx43 derivatives synthesized by cell-free protein synthesis in the presence of 10% nickel-chelating Rho-DMPE liposomes.

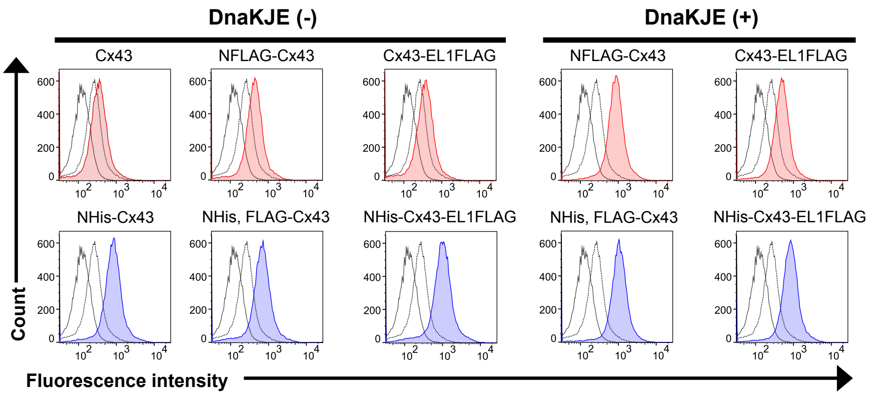


**Figure S4. Detection of C-terminal domain of Cx43 derivatives.** Flow cytometric analysis of Cx43 derivatives-integrated proteoliposomes (red area) and NHis-Cx43 derivatives-integrated proteoliposomes (blue area) using Cx43-specific antibody. Solid lines indicate the loading of Cx43-specific antibody modified beads and dash lines indicate the loading of beads reacted with His-KcsA-integrated proteoliposomes.

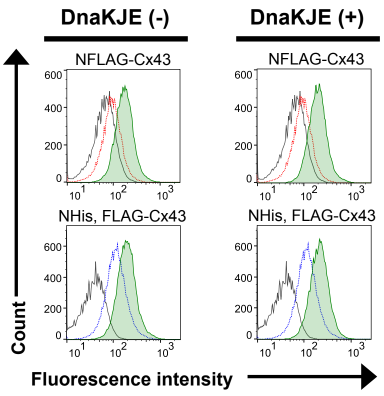


**Figure S5. Detection of N-terminal domain of NFLAG-Cx43 and NHis, FLAG-Cx43.** Flow cytometric analysis of NFLAG-Cx43 and NHis, FLAG-Cx43-integrated proteoliposomes (green area) using FLAG-tag-specific antibody. Solid lines indicate the loading of FLAG-tag-specific antibody modified beads. Red and blue lines indicate the loading of beads reacted with Cx43-integrated proteoliposomes and NHis-Cx43-integrated proteoliposomes, respectively.

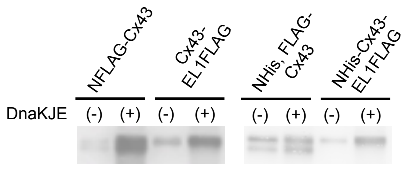


**Figure S6. Increase in solubility of Cx43-FLAG fusion proteins by molecular chaperon DnaKJE.** Western blot analysis of solubilized Cx43-FLAG fusion proteins synthesized by cell-free protein synthesis in the presence of 10% nickel-chelating Rho-DMPE liposomes with or without DnaKJE. Cx43-FLAG fusion proteins-integrated proteoliposomes (40 pmol lipids) and NHis-Cx43-FLAG fusion proteins-integrated proteolliposomes (20 pmol lipids) were subjected to western blot analysis by using Cx43-specific antibody.

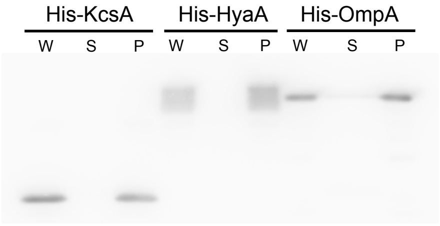


**Figure S7. Aggregation-prone His-KcsA, His-HyaA and NHis-OmpA synthesized without liposomes.** Western blot analysis of His-KcsA, His-HyaA and NHis-OmpA synthesized using cell-free protein synthesis in the absence of liposomes. The expressed whole sample (W) was ultracentrifugated, and collected supernatants (S) and pellets (P).

**References**

S1) Ando, M.; Akiyama, M.; Okuno, D.; Hirano, M.; Ide, T.; Sawada, S.; Sasaki, Y.; Akiyoshi, K., Liposome chaperon in cell-free membrane protein synthesis: one-step preparation of KcsA-integrated liposomes and electrophysiological analysis by the planar bilayer method. *Biomaterials Science* **2016,** *4* (2), 258-264.

S2) Hirokawa T.; Boon-Chieng S.; and Mitaku S.; SOSUI: classification and secondary structure prediction system for membrane proteins. *Bioinformatics* 1998, 14 378-379
